# Pesticide exposure and human health: Toxic legacy

**DOI:** 10.1016/j.clinsp.2023.100249

**Published:** 2023-07-20

**Authors:** Fulvio A. Scorza, Larissa Beltramim, Larissa M. Bombardi

**Affiliations:** a Ministério do Desenvolvimento Agrário e Agricultura Familiar (MDA), Brasília, DF, Brazil; b Disciplina de Neurociência, Escola Paulista de Medicina, Universidade Federal de São Paulo (EPM/UNIFESP), São Paulo, SP, Brazil; c Faculdade de Filosofia, Letras e Ciências Humanas, Universidade de São Paulo (USP), São Paulo, SP, Brazil

In the last decades, agriculture had started to take on a global scale, not only in the sense that a significant part of the agricultural production started to be globally commercialized but also because it began to become dependent on the (synthetic) chemical industries producing patented fertilizers, pesticides and seeds.^1^, ^2^, ^3^ In these terms, the justification for the industrialization of agriculture was the promise of overcoming hunger, through the use of technology.^1^, ^2^, ^3^ Unfortunately, more than half a century has passed since the inception of “Green Revolution” technologies and world hunger persists.^1^, ^2^, ^3^ Whilst global hunger has increased, humans and their environment have been intensely contaminated by synthetic chemical substances used in agriculture.^1^, ^2^, ^3^ In fact, between 2010 and 2021, the use of pesticides in Brazil substantially increased by 91% (IBAMA 2010‒2020).^4^ As a consequence of this increase, the authors are witnessing harmful chemical violence, which arises as an unfolding of the aforementioned Green Revolution.^1^^,^^2^ Thus, the authors may call this form of violence “Chemical Colonialism”, since substances banned for more than ten or twenty years in the European Union because of risks to human and environmental health continue to be sold indiscriminately – by companies headquartered in the European Union – to countries such as Brazil.^1^

It is widely known that these substances bring severe impacts to human health and the environment.^1^^,^^2^ Suffice it to say that in the early 1990s, the World Health Organization (WHO) estimated that around one million people were involuntarily intoxicated with pesticides annually.^1^, ^2^, ^3^ Actually, the number is 385 million unintentional poisonings, of which 11,000 are fatal.^1^, ^2^, ^3^ In this context, Boedeker and colleagues indicate that pesticide poisoning is a very serious problem, widespread worldwide and requiring immediate action.^3^

Considering these data, recent Brazilian research suggests that pesticides have detrimental effects on human health.^5^ Unfortunately, several negative health effects are related to chemical pesticide exposure such as dermatological, gastrointestinal, respiratory, reproductive, endocrine, child growth, neurological, and carcinogenic effects.^6^, ^7^, ^8^ For example, it has been clearly shown that absorption resulting from dermal pesticide exposure is the main route of uptake for exposed subjects.^9^^,^^10^ In this sense, occupational and residential use of pesticides has been linked to cutaneous melanoma in epidemiological studies.^10^ Furthermore, as pesticides are often ingested as residues in food commodities and water, it has been described that pesticides can disrupt the typical composition and functionality of gut microbiota, leading to many diseases in humans.^11^^,^^12^ Interestingly, as human exposure to pesticides can occur in the workplace, in the household, and through the ambient environment, a number of studies have evaluated the impact of pesticide exposure on human respiratory health.^13^^,^^14^ Thus, it has been found that occupational pesticide exposures are associated with self-reported coughing, wheezing, and airway inflammation, asthma, chronic obstructive pulmonary disease, lung cancer, and impaired lung function.^13^^,^^14^ Moreover, other studies have also evaluated the possible action of pesticides on the endocrine system and its functions.^15^^,^^16^ In these lines, experimental studies indicate that exposure to high doses of pesticides modify endocrine function, disrupting gonadotropin, thyroid, and sex-steroid hormone signaling and inducing oxidative stress altering sexual development, fecundity, and maintenance of pregnancy.^15^ At the human level, it has been found that the excessive use of synthetic pesticides disrupts reproductive and sexual development, and these effects seem to depend on several factors, including gender, age, diet, and occupation.^16^ Moreover, epidemiological studies have reported that exposure to pesticides may be linked with menstrual cycle disturbances, reduced fertility, prolonged time-to-pregnancy, spontaneous abortion, stillbirths, and developmental defects, which may or may not be due to disruption of the female hormonal function.^17^ Importantly, children's exposures to pesticides should be limited as much as possible since human fetuses, infants, and children show greater susceptibility than adults.^15^^,^^16^^,^^18^^,^^19^ In addition, other studies have associated parental pesticide use and adverse birth outcomes including physical birth defects, low birth weight, and fetal death.^18^ Furthermore, in recent years, several studies have demonstrated that parents or their children occupationally exposed to pesticides have a higher risk of presenting difficulties in their neurobehavioral, neurocognitive or neuromotor performance.^20^^,^^21^ Unfortunately, important studies also identified that some pesticide families (i.e., carbamates, organochlorines and organophosphates) can cause serious damage to the nervous system and are a risk factor for developing Parkinson's Disease (PD).^22^ Worryingly, it was also shown that the 5 and 10 years of duration of pesticide exposure were associated with a 5% and 11% augment in the risk of PD.^22^ With regard to cancer, the scenario is very similar. Thus, a group of Brazilian scientists conducted an integrative literature review of published studies on pesticide and cancer exposure, focusing on farmers, rural populations, pesticide applicators, and rural workers.^23^ The most consistent associations found were prostate cancer, Non-Hodgkin lymphoma, leukemia, multiple myeloma, bladder and colon cancers.^23^ In parallel, studies that further investigate the relationship between pesticides and neoplasms of the testis, breast, esophagus, kidney, thyroid, lip, head/neck, and bone are also recommended.^23^ In this sense, oncology researchers believe that there is enough evidence to recommend that patients reduce exposure to all pesticides.^24^

Following these lines of reasoning line, this scenario demonstrates the importance of addressing the topic “pesticides and human health” on the public agenda and the urgency to intensify the coordination and articulation of public policies in the area of agriculture, agrarian development, and family farming, considering its interfaces with public policies in the economic, infrastructure, social and environmental areas. Obviously, such actions must be based on sovereignty, the principle of human dignity, and fundamental rights provided for by law.^25^

Overall, the authors are totally in agreement that the scientific community has been working hard to come up with creative approaches to pesticide pollution reduction.^13^ Furthermore, the authors also believe that environmentally friendly management strategies which include some bioremediation approaches (i.e., phytoremediation, microalgae bioremediation, mycoremediation, and microbial degradation) are viable ecological alternatives.^13^ Finally, new considerations and experimental, epidemiological, and clinical studies should be carried out to establish with precision how pesticide exposures affect human health. Meanwhile, caution with pesticide exposure remains prudent and necessary.

## Declaration of Competing Interest

The authors declare no conflicts of interest.
